# Rhabdomyosarcome paratesticulaire chez un nourrisson de 14 mois

**DOI:** 10.11604/pamj.2014.17.24.2233

**Published:** 2014-01-17

**Authors:** Khalid Khattala, Youssef Bouabdallah

**Affiliations:** 1 Service de chirurgie pédiatrique, CHU Hassan II, Fès, Maroc

**Keywords:** Rhabdomyosarcome, testicule, nourrisson, rhabdomyosarcoma, testicle, infant

## Image en medicine

Le rhabdomyasarocme paratesticulaire est une tumeur maligne rare, avec deux pics de fréquence à l’âge de 5 ans et un autre à 16 ans, le diagnostic repose sur l’échographie, la TDM et IRM et confirmé par l’étude anatomopathologique, le traitement repose sur l'association d'une chimiothérapie et d'une exérèse chirurgicale complétée par une radiothérapie sur les foyers résiduels et sur les ganglions rétropéritonéaux. Nous rapportons le cas d'un nourrisson de 14 mois qui présente depuis la naissance une petite masse d'un demi centimètre au niveau scrotal droit, qui a augmenté progressivement et n'a consulté que lorsque la masse est devenue énorme, le bilan radiologique a objectivé une masse scrotal sans autres localisations, il a bénéficié d'une hemiscrotectomie droite, avec le diagnostic d'un rhabdomyosarcome para testiculaire à l’étude histologique, malheureusement il n'a pas pu bénéficié d'une chimiothérapie faute de moyens, 2 mois après, il est revenu pour une récidive locale, repris chirurgicalement, le malade est décédé 2 mois en postopératoiore dans un tableau d'aplasie médullaire.

**Figure 1:**
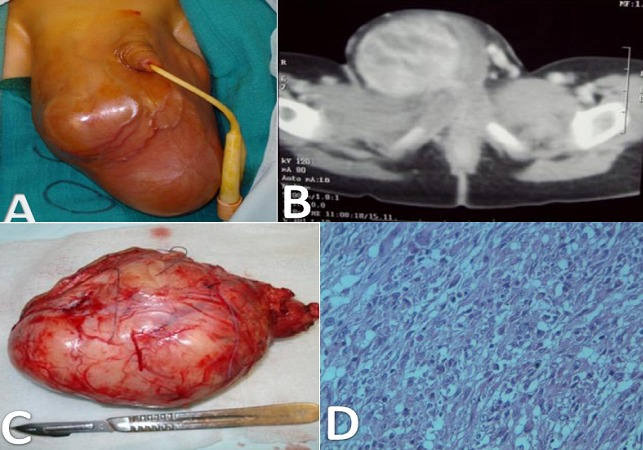
A) grosse bourse droite, sans signes inflammatoires en regard, avec à l'examen une masse accolée au testicule; B) coupe scanographique axiales montrant une volumineuse masse sans visualisation du testicule homolatéral; C) tumeur blanche, luisante encapsulée mesurant 15 cm de long; D) coupe microscopique montrant un rhabdomyosarcome de type embryonnaire à cellules fusiformes

